# Deep-UV photoinduced chemical patterning at the micro- and nanoscale for directed self-assembly

**DOI:** 10.1038/s41598-018-28196-1

**Published:** 2018-07-11

**Authors:** Benjamin Leuschel, Agnieszka Gwiazda, Wajdi Heni, Frédéric Diot, Shang-Yu Yu, Clémentine Bidaud, Laurent Vonna, Arnaud Ponche, Hamidou Haidara, Olivier Soppera

**Affiliations:** Institut de Science des Matériaux de Mulhouse, CNRS-UMR 7361, Université de Haute Alsace, 15 rue Jean Starcky, 68057 Mulhouse, France

## Abstract

Deep-UV (DUV) laser patterning has been widely used in recent years for micro- and nanopatterning, taking advantage of the specific properties of irradiation with high-energy photons. In this paper, we show the usefulness of DUV laser patterning for preparing surfaces with controlled chemical properties at the micro- and nanoscale. Our motivation was to develop a simple and versatile method for chemical patterning at multiscales (from mm to nm) over relatively wide areas (mm^2^ to cm^2^). The chemical properties were provided by self-assembled monolayers (SAMs), prepared on glass or silicon wafers. We first investigated their modification under our irradiation conditions (ArF laser) using AFM, XPS and contact angle measurements. Photopatterning was then demonstrated with minimum feature sizes as small as 75 nm, and we showed the possibility to regraft a second SAM on the irradiated regions. Finally, we used these chemically patterned surfaces for directed self-assembly of several types of objects, such as block copolymers, sol-gel materials and liquids by vapor condensation.

## Introduction

Micro- and nanofabrication are key steps for any significant advance in nanoscience and nanotechnology. However, achieving efficient, easy and reproducible nanopatterning processes for fundamental and practical applications has always been a major challenge in nanotechnology^1,2^. Driving most of the innovation in this field, the microelectronic industry has, in recent decades, devoted much effort to developing new processes and the associated materials to obtain a progressive decrease in the sizes of devices, primarily via photolithography processes^3^. The use of deeper UV wavelengths in combination with immersion lithography or other strategies, such as double patterning, have been proposed and industrialized to fulfil the requirements of the silicon industry. These technologies, so called “top-down” techniques, have reached a very high level of maturity, suitable for fabrication of sophisticated devices with high quality control. However, such performances are only achievable at considerable cost, and these techniques are well-adapted only for silicon-based devices. Alternative techniques derived from photolithography, such as e-beam^4^, X-ray^5^, optical near-field^6,7^ or scanning probe lithography^8^, have also been developed but remain restricted to narrow fields of applications, primarily due to their very low throughputs.

In parallel to top-down techniques, bottom-up approaches bring many advantages, including the use of well-defined nanoscale building blocks and their organization by spontaneous processes, leading to low-cost processes that can provide smart solutions for specific applications. However, the main remaining challenge of these bottom-up techniques is to achieve controlled deposition of these nano-objects in well-defined positions and regular organization over wide surfaces. These objectives correspond to those of directed self-assembly (DSA), and many efforts have been devoted to developing original methods for controlled self-assembly of nanoparticles^9^, block copolymers^10,11^ and proteins, for example.

Basically, DSA relies on the use of patterned substrates with typical dimensions on the same order of magnitude as the objects to be deposited on. This patterning can be achieved by two means: topographical (grapho-epitaxy) or chemical (chemo-epitaxy) contrast. In the first case, relief patterns are built on the substrate, and the ridges are used to guide the self-assembly process. This route is particularly useful for block copolymer self-assembly^12,13^. One drawback of grapho-epitaxy is the generation of uneven surfaces, which may be a limitation for applications requiring flat surfaces. The second option consists of achieving deposition on chemically controlled surfaces (chemo-epitaxy). Self-assembly can indeed be driven by the local chemical affinity of the surface. Many different processes have been developed to achieve chemical patterning at the micro- and nanoscale in this context. These methods are primarily based on one of the following approaches: a photoresist is initially patterned and then used to block the grafting of molecules in defined regions. Self-assembled monolayers (SAMs) prepared from silanes or thiols (for Si or Au substrates, respectively) are usually used. The photoresist can then be stripped, and a second SAM can be grafted in regions where the substrate remains free of functionalization. The main drawback of this technique is the many experimental steps that it requires. For this reason, direct patterning of SAMs has been proposed as an alternative method. In this case, the SAM is grafted throughout the entire surface and is directly modified by exposure to spatially controlled radiation. Radiation can be used to photolyze the SAM or to graft new functional groups on the molecule ends, as illustrated in^14^.

Light has been widely used for patterning organosilane monolayers. Dulcey *et al*. showed that SAMs can be patterned with deep-UV (DUV) light^15^. In this example, the resolution of patterns is limited by diffraction. In other works, they proposed the use of phenyl, naphthyl and anthracenyl precursors for DUV photopatterning because of the strong absorption of the phenyl groups for the DUV wavelengths^16–19^. Another approach to modifying the nature of an SAM with spatial control has been proposed by Whitesides *et al*.^20^ through the use of multiple wavelength irradiation to obtain spatially controlled patterns with different chemistries. With this approach, the photocleavage of SAMs has been demonstrated for an SAM on gold substrates. The maximum resolution is on the order of 10 microns due to the mask fabrication method.

High resolution SAM patterning has been achieved using electron beam lithography. Patterns with typical dimensions of less than 100 nm were obtained with this technique^21,22^. However, e-beam lithography has significant drawbacks regarding a need for very expensive instrumentation and long fabrication times if cm^2^ surfaces are required. Likewise, X-ray lithography was proposed as a method for SAM patterning^23^.

Furthermore, alternative patterning methods relying on scanning probe lithography have been proposed: dip-pen lithography^24,25^, electrochemical modification of SAMs^26^, nanoshaving^27^ and bias-induced lithography^28^ allow chemical patterning with nanoscale resolution, but these methods suffer from a lack of reproducibility and are highly time consuming, which has thus far prevented them from being used in manufacturing.

Soft lithography^29,30^ can be used to transfer organosilanes from PDMS molds to glass or Si substrates. Nanopatterning can be achieved with PDMS molds with nanoscale features, but in this case, the fabrication of the primary mask is complex, and the risk of defects appearing during transfer causes issues. Similarly, precise alignment of PDMS stamps is challenging for practical applications.

The objective of this paper is to report on recent works conducted by our research team in the framework of directed self-assembly using DUV laser patterning. Our motivation is to develop a relatively simple and versatile method for chemical patterning of SAMs at multiscales (from mm to nm) over relatively wide regions (typically several cm^2^). As a final goal, we aim to show that this approach is suitable for DSA of several types of objects, such as block copolymers, nanoparticles or liquids. Among other micro- and nanofabrication approaches developed by our team, DUV patterning (λ = 193 nm) has been widely used in recent years for microelectronic photoresist studies^31,32^, DSA of block copolymers by grapho-epitaxy^33,34^, nanopatterning of polymer surfaces for biological applications^35,36^ and metal-oxo cluster micro- and nanopatterning^37–40^.

The choice of DUV laser irradiation (an ArF excimer laser emitting at 193 nm) for SAM patterning is based on the following reasons:

(i) The energy of 193 nm photons (148 kcal/mol) exceeds the energy of the Si-C bonds in silanes used to prepare the SAM (88 kcal/mol). Bond breakage is thus expected without the need to design specific functions in the molecular structure. Accordingly, any silane can potentially be patterned with this wavelength. The use of a sacrificial photoresist is therefore unnecessary, which greatly facilitates the process.
(ii) DUV light is strongly absorbed by organic moieties, so significant light absorption can be achieved even for the extremely thin films formed by SAMs (thicknesses of 1 to 2 nm), which limits the irradiation time to reasonable values compatible with practical applications. It must be noted that attempts with the same procedure with a frequency-quadrupled Nd:YAG laser (emitting at 266 nm) showed no possibility of patterning the SAM in equivalent conditions (power, time), indicating that wavelengths shorter than 200 nm are required.
(iii) Low wavelength light is associated with high resolution patterning because the diffraction limit is directly linked to the wavelength.
(iv) Compared to DUV lamps, lasers deliver higher intensity and avoid radiative heating of the sample, and the coherence of laser light opens the door towards patterning by interferometric methods, which is a very convenient technique for generating high resolution patterning of periodic 1D and 2D structures with a single exposure. This gives a significant advantage over e-beam or scanning probe lithography for patterning over wide regions. Even though it is a light-based method, high resolution patterning is possible, with resolution below 50 nm^41^. The use of VUV (λ = 157 nm) or EUV lithography (λ = 13.4 nm) has been proposed to improve the resolution in interferometric configurations, but these techniques require sophisticated irradiation systems and the use of vacuum chambers, as these wavelengths are absorbed by air, which makes them less convenient^42–45^. Whereas previous works primarily focused on alkenylsiloxanes and aminosiloxanes, we used alkylsiloxane SAMs for the high versatility associated with the wide choice of commercially available silane derivatives. The relatively low absorption of such alkylsiloxanes at 193 nm can be compensated by increasing the laser power and, if higher patterning speeds are required, focusing the beam onto the substrate to increase the local energy density.

**Figure 1:**
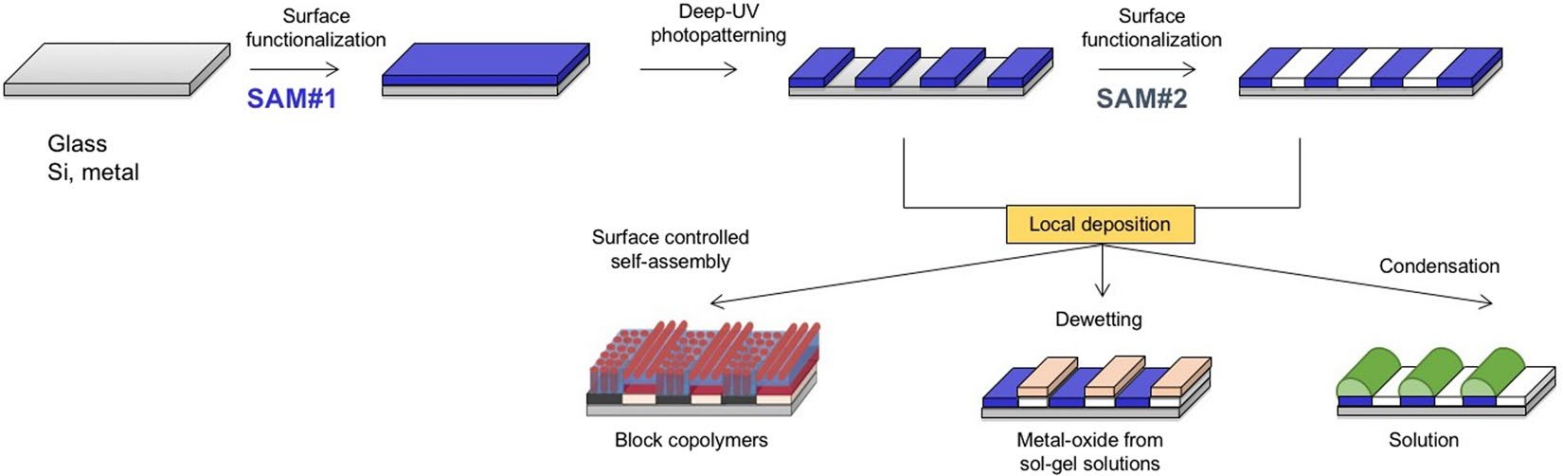
Schematic representation of the DUV-DSA approach. First row shows the surface functionalization by SAM#1, the local DUV patterning and the possibility of regrafting in the irradiated zones by SAM#2. Second row illustrates the use of micro- and nanopatterned surfaces for directed self-assembly of block copolymers induced by surface chemistry, metal-oxide patterning by dewetting of precursor solutions and spatial control of liquid condensation.

In the first section, we present the DUV modification of SAMs, investigated using AFM, XPS and contact angle measurements. Photopatterning is demonstrated with minimum feature sizes as small as 75 nm, and we show the possibility to regraft a second SAM on the irradiated regions, as schematized in Fig. 1. The second section is aimed at illustrating the usefulness of this approach, providing applications for micro- and nanopatterning of block copolymers, functional sol-gel solutions and liquids.

## Experimental

### Self-Assembled Monolayers

Preparation of self-assembled monolayers (SAMs) was conducted following a classical procedure, described in previous studies^46–48^. Silicon wafers were used as substrates. In some specific cases, SiN and glass were used. The substrates were first cleaned by UV-ozone irradiation (Bioforce Procleaner plus) or immersion in a piranha solution (3:7 (v:v) mixtures of 30% H_2_O_2_/H_2_SO_4_, thermostated at 50 °C for 30 minutes), then thoroughly rinsed with de-ionized water and dried under nitrogen flow. Warning: Piranha solutions are extremely dangerous and may result in explosion or skin burns if not handled with extreme caution. This step also leads to a high coverage of silanol groups on the silicon surface, to which silanes can bind and self-assemble through condensation reactions with the substrate and van der Waals interactions between aliphatic chains.

**Figure 2:**
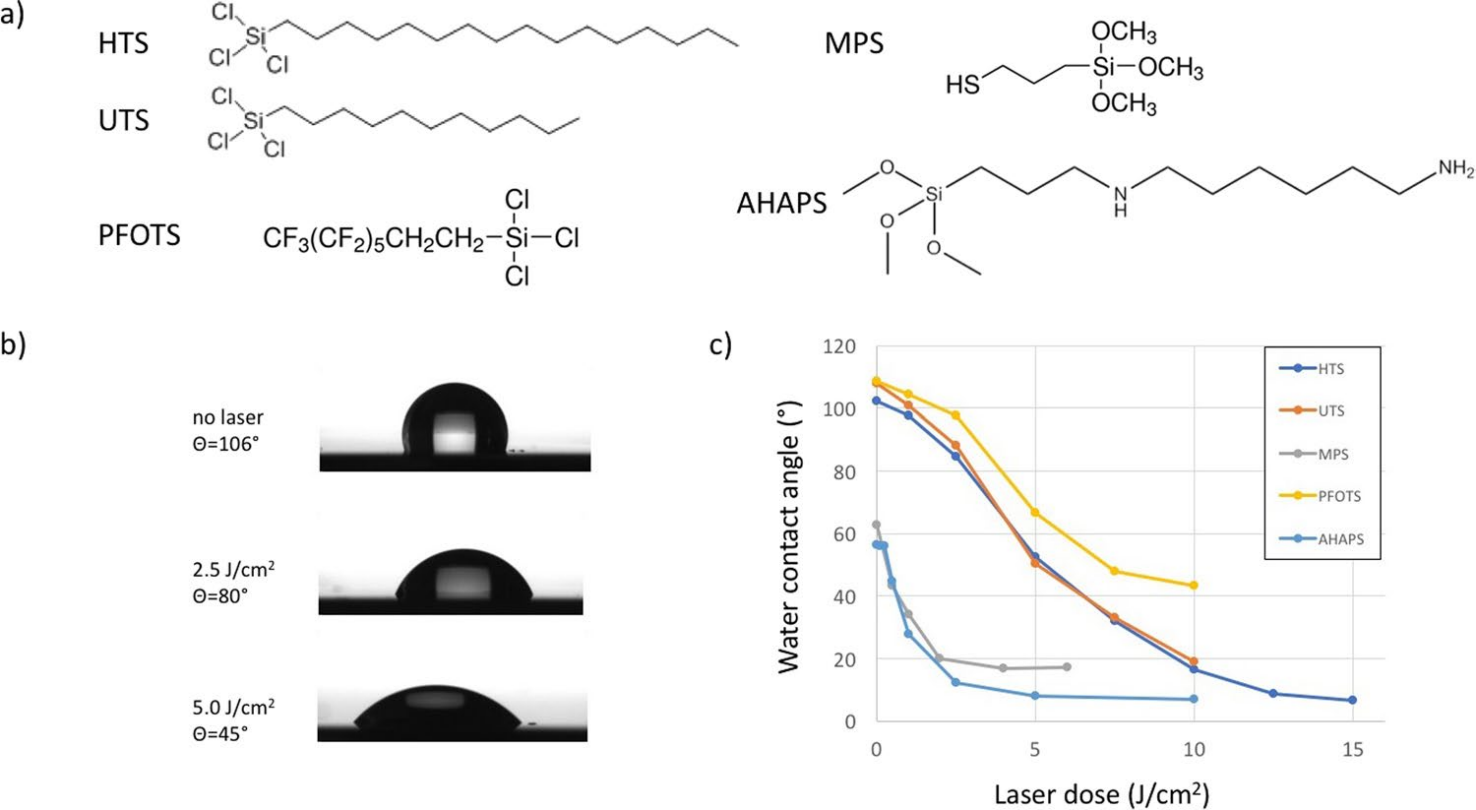
(**a**) Molecular structures of the silanes used in this study. (**b**) Images of water drops deposited on HTS surfaces before and after DUV irradiation at 2.5 and 5 J/cm^2^. (**c**) Modification of the water contact angle by DUV irradiation for HTS, UTS, MPS, PFOTS and AHAPS on Si substrates.

The freshly prepared surfaces were then immersed in silane precursor solutions overnight. HTS (hexadecyltrichlorosilane, 95%, ABCR GbmH), UTS (undecyltrichlorosilane, 97%, ABCR GbmH), MPS ((3-mercaptopropyl)trimethoxysilane, 95%, Sigma Aldrich), PFOTS (trichloro(1 H,1 H,2 H,2H-perfluorooctyl)silane, 97%, Sigma Aldrich) and AHAPS (N-(6-aminohexyl)-aminopropyltrimethoxysilane, 92%, ABCR GbmH) were used as received. Molecular structures are given in Fig. 2a. HTS and UTS solutions (1 mM) were prepared in chloroform/cyclohexane (50/50 vol. %), and MPS solution (3 mM) was prepared in toluene. PFOTS solution (1 mM) was prepared in chloroform. AHAPS (1 mM) was prepared in anhydrous ethanol. After the reactions, the substrates were carefully rinsed to eliminate any remaining ungrafted precursors. Chloroform (anhydrous, ≥99%), Aldrich, cyclohexane (anhydrous, 99.5%), Aldrich, toluene (anhydrous, 99.8%) and ethanol (anhydrous) were provided by Sigma-Aldrich.

### DUV irradiation

Photoinduced modification of SAMs was conducted via deep-UV irradiation using an excimer laser emitting at 193 nm (Braggstar from Coherent). The typical power was 28 mW (pulse repetition rate of 20 Hz). This laser was used either for homogeneous irradiation with the raw laser beam (1 cm^2^ irradiated area) or for patterning. In the latter case, two setups were used: for lowest resolution patterning (from 0.8 to 10 microns), binary masks consisting of metal lines on fused silica substrates were used. To achieve higher resolution, interference lithography with phase masks was used. Details of these experimental configurations can be found in^38^. Depending on the period of the phase masks, patterns with widths ranging from 75 nm to 300 nm could be produced.

### Atomic Force Microscopy (AFM) imaging

The AFM equipment used here is a Picoscan system from Molecular Imaging. It was primarily used in contact mode to record friction images (lateral deflection). In this case, AFM contact mode probes with a typical stiffness of approximately 0.2 N/m were used (Contact-G tips from BudgetSensors). Scanning was performed at 1 line/sec with an image resolution of 512 × 512 pixels. Friction measurements were very effective in revealing chemical contrast. Unfortunately, the friction measurement could not be used quantitatively because the recorded data depend on many parameters related to the tip surface and geometry, applied force, scanning speed, etc.

Phase images of block copolymer surfaces were recorded using resonant AFM. In this case, Si_3_N_4_ tips with a typical resonance frequency of 285 kHz (stiffness 42 N/m, ACT tips from AppNano) were used. Scanning was performed at 0.5 line/sec with an image resolution of 512 × 512 pixels.

### Contact angle

Contact angle measurements were performed with an automated goniometer from Krüss (DSA 100, Germany) using 2 µl drops of demineralized water.

### XPS

XPS analysis was performed on a Gammadata Scienta (Uppsala, Sweden) SES 200-2 X-ray photoelectron spectrometer under ultra-high vacuum (P < 10^−9^ mbar). The monochromatized AlKa source (1486.6 eV) was operated at a power of 420 W (30 mA and 14 kV), and the spectra were acquired at a take-off angle (TOA) of 90° (angle between the sample surface and photoemission direction). During acquisition, the pass energy was set to 500 eV for wide scans and to 100 eV for high-resolution spectra. CASAXPS software (Casa Software Ltd, Teignmouth, UK, www.casaxps.com) was used for all peak fitting procedures, and the areas of each component were corrected according to classical Scofield sensitivity factors (Si2p: 0.817, C1s: 1.00 and O1s: 2.93) and the transmission function of the spectrometer. All components on high-resolution spectra were referenced according to the CH_x_ component at 285.0 eV.

The reproducibility of atomic quantification obtained on a Scienta spectrometer is estimated to be less than 10%. It is also worth noting that due to the low thickness of the films, there is no need for charge compensation in our system, and consequently, degradation occurring in the organic film during an XPS experiment is limited. Because the XPS experiments are time consuming, we could not reproduce each piece of data several times. Only the HTS-grafted surface was characterized several times because this chemistry was used in different applications.

### Block copolymer thin films

The diblock copolymer used in this study was PS-b-PMMA, (43000 and 21000 g/mol) provided by Polymer Source Inc. It was dissolved in toluene at a concentration of 5 mg/ml. This polymer self-assembles into cylinders of PMMA in a PS matrix with a period of 35 nm^34^. Thin films were prepared by spin-coating (Suss Microtec Delta). Film thicknesses were adjusted by varying the rotation speed between 2500 and 5000 rpm and were measured using spectroscopic ellipsometry (Horiba-Jobin Yvon).

### Sol-gel solution

The 0.25 M sol-gel InO solution was prepared by dissolving indium nitrate hydrate (In(NO_3_)_3_ ⋅ H_2_O, Sigma-Aldrich) and methoxyethanol (MOE) (molar ratio In:MOE = 1:2) into 1-propanol. The solution was aged under stirring for 24 hours at room temperature to obtain a stable homogeneous solution.

The sol-gel IGZO solution was prepared by dissolving indium nitrate hydrate (In(NO_3_)_3_ ⋅ H_2_O, Sigma-Aldrich), gallium nitrate hydrate (Ga(NO_3_)_3_ ⋅ H_2_O, Sigma-Aldrich), and zinc methacrylate (Zn(CH_2_CH_3_COO)_2_, Sigma-Aldrich) precursors in 2-methoxyethanol (CH_3_OCH_2_CH_2_OH, Sigma-Aldrich). The molar ratio of In, Ga, and Zn was 4:1:2, and the total molar concentration of metals was 0.25 M. All solutions were aged under stirring for 24 hours at room temperature to obtain stable homogeneous solutions.

Solutions were filtered (Teflon filters with 0.2 μm pores) and deposited on freshly chemically patterned substrates via spin-coating. Thermal annealing (80 °C, 1 hour) was conducted to remove completely the solvent and crosslink the structures.

## Results and Discussion

### Tailoring the surface chemistry through DUV irradiation

This first section is aimed at demonstrating the modification of the surface chemistry under DUV irradiation and investigating the involved physico-chemical molecular phenomena.

Five different methoxysilanes or chlorosilanes bearing different functional chains were used in this study (Fig. 2a). The SAMs were first characterized by measuring the equilibrium contact angle with water. For each SAM, five samples were prepared, and for each sample, five measurements were conducted in different regions to check the reproducibility of the SAM preparation. On Si substrates, the standard deviation was typically 1° for all SAMs before irradiation, demonstrating that our preparation procedure is reproducible. Moreover, the contact angles with water correspond exactly with the expected values from refs^46–48^. AFM topography images were also systematically recorded, and we observed a flat surface at the molecular scale, confirming the structures of the SAMs.

In the case of HTS, the equilibrium contact angle with water was 106° after SAM preparation, which is in good agreement with the expected value for a close-packed monomolecular film.

The contact angle of the HTS surface decreased continuously with increasing DUV irradiation dose, as illustrated in Fig. 2b. Above 10 J/cm^2^, the contact angle with water corresponds to a hydrophilic surface. We noted that for higher doses (>10 J/cm^2^), the standard deviation increased greatly (5°) due to the difficulty in measuring small contact angles. As a consequence, for contact angles below 15°, we could not record any significant differences, and we considered a complete wetting of the surface.

Figure 2c shows the decrease in the contact angle with water for the different SAMs. Interestingly, the behaviors of HTS and UTS are quite similar, showing that the initial alkane chain length has a poor influence on the photoinduced modification as soon as conditions to obtain a close-packed assembly are guaranteed.

For silanes bearing thiol or amine groups, a much faster photodegradation was observed. Degradation was obtained for 2.5 J/cm^2^, compared to 10 J/cm^2^ for alkylsilanes. This faster rate can be attributed to the higher oxidation potential of thiol and amine functions compared to alkane chains. In addition, it is generally admitted that these precursors can hardly give rise to close-packed molecular arrangements. Thus, more defects are present in the SAM film, which may support the photodegradation of the layer, as already suggested in previous works^18,48^.

Finally, we illustrate that an adjustment of the contact angle with an SAM prepared from fluorinated precursors (PFOTS) is also possible. This possibility is quite useful for achieving surfaces with tunable polarity contrast over a wide range.

**Figure 3:**
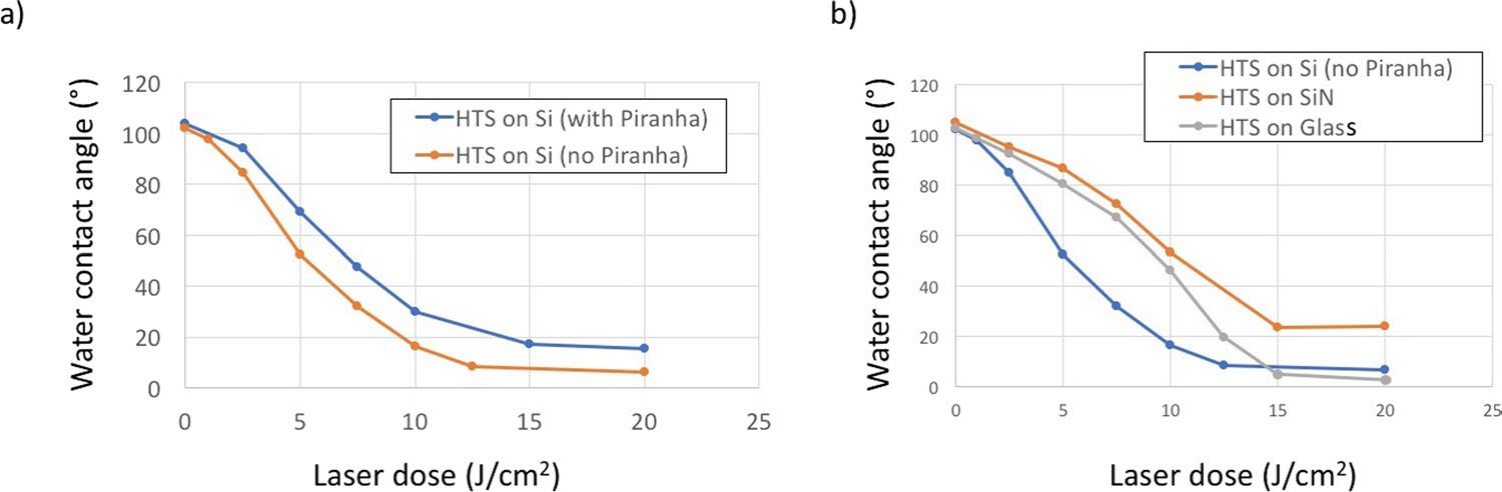
(**a**) Modification of the water drop contact angle of HTS surfaces with DUV irradiation for an SAM prepared with and without piranha treatment. (**b**) Influence of the nature of the substrate (Si, SiN and glass) on the DUV modification of the HTS SAM.

Interestingly, two additional parameters were shown to be very important for controlling the photodegradation of the SAM: (i) the substrate activation process used to graft the SAM and (ii) the nature of the substrate. In (Fig. 3a**)**, we compare the DUV-induced modification of HTS SAM on silicon with substrates cleaned and activated with piranha or UV-ozone cleaner prior to the grafting process. We observe that photodegradation is slower when a piranha treatment is used. This result shows that the structure of the molecular film has an effect on the rate of photolysis. Indeed, immersion of Si substrates in a piranha solution is known to be the most effective process for activating surface SiOH functions.

It is reported that close-packed and ordered SAMs can easily be obtained on silicon substrates that are characterized by an optimal surface density of reactive silanol groups (5 SiOH/nm²) after proper cleaning and prehydration, which is usually obtained by piranha treatment. Additionally, the extremely low roughness of the silicon wafer compared to that of a glass substrate is a second fundamental parameter that contributes to the close packing and high ordering of the monolayer. Under these conditions, very clean and reactive surfaces can be obtained, and thus, the quality of the SAM is improved with an optimum molecule density corresponding to a close-packed layer, which considerably slows down the photodegradation kinetics compared to monolayers with more defects. As a consequence, the quality of the SAM can be used as a parameter to tune the photoreactivity of the molecular layer. Considering the conditions to prepare the surface, the contact angles with water and the AFM images, we assume that we have close-packed SAM layers. FTIR spectroscopy and ellipsometry have been proposed by other groups to confirm the structure of SAMs^49,50^.

As shown in (Fig. 3b), the nature of the substrate also has a strong effect on the reactivity. Chemically modified Si substrates were found to be twice as efficient as glass or SiN. This difference is probably due to the strong reflection of DUV light by Si, in contrast to glass and SiN. Thus, the radiation received by the molecular layer is approximately twice the incident radiation dose on Si, whereas SiN absorbs DUV light and avoids this reflection effect. Interestingly, glass substrates showed kinetics similar to that of SiN at low irradiation doses, but then, acceleration was observed. The higher roughness of the glass substrates in comparison to SiN and Si may account for this behavior, as it increases the appearance of defects in the monolayer, which could favor the degradation of the SAM, as previously explained.

To further investigate the photoinduced phenomena and confirm the local effects of DUV radiation on the HTS layer, we performed XPS measurements of the HTS monolayer before and after irradiation with increasing doses.

**Figure 4:**
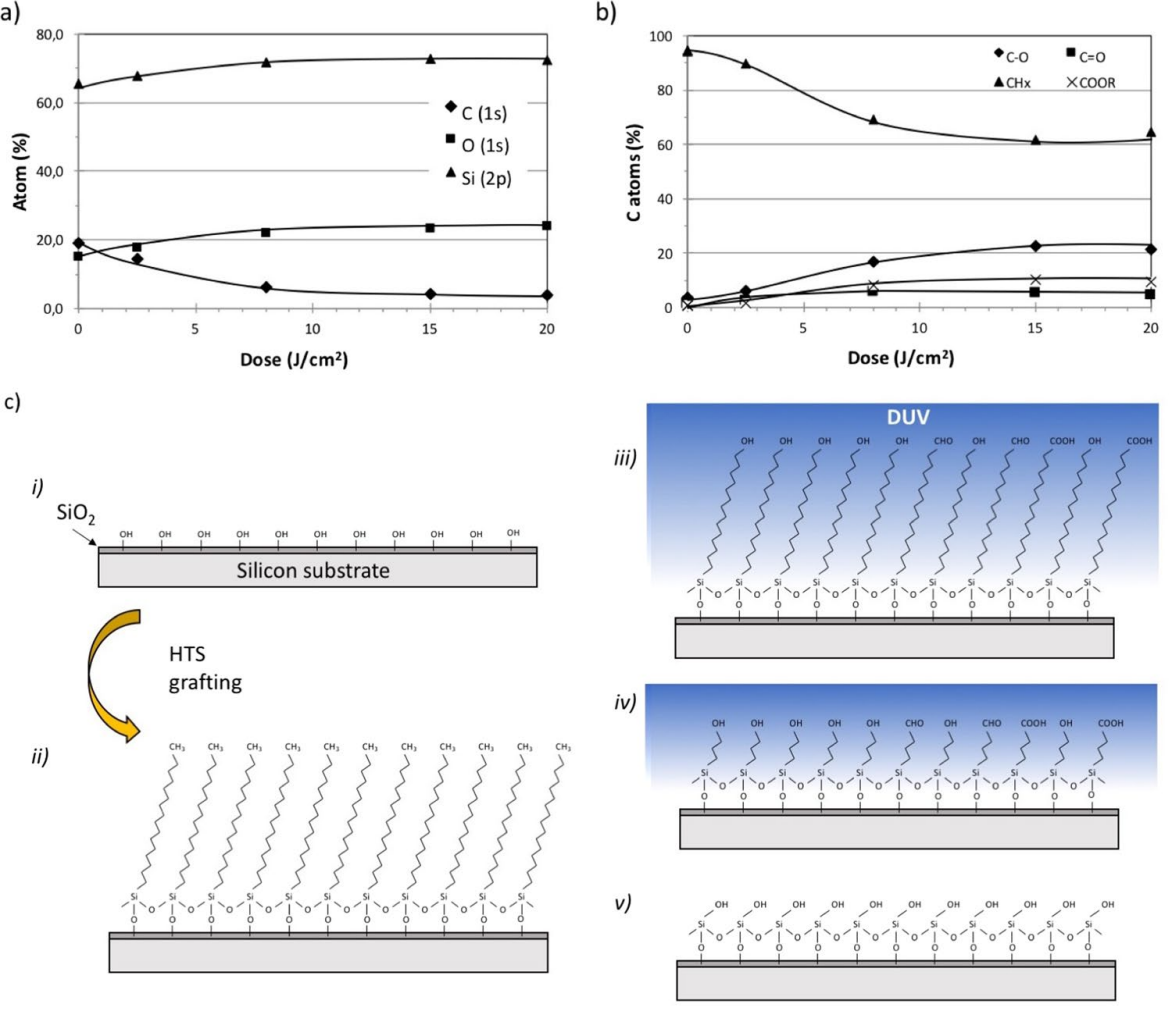
XPS analysis of HTS monolayers under DUV irradiation. (**a**) Atomic composition following the proportions (in %) of C, O and Si. (**b**) C(1 s) different species proportions determined from deconvolution of the C(1 s) peak. (**c**) Schematic view of the proposed DUV-induced degradation mechanism: (i) schematization of the Si substrate with it native oxide thin layer; (ii) the Si wafer grafted with HTS; (iii) the partially oxidized HTS SAM layer due to DUV irradiation; and (iv) cleavage of the alkyl chains, leading finally to (v) the regeneration of a SiOH surface.

The atomic composition of the surface was extracted from XPS spectra (raw spectra are given in SI1) for different doses of DUV radiation (Fig. 4a). For each dose, a new sample was prepared. The regular modification of the XPS spectra with irradiation dose shows that the data obtained by XPS are consistent. A significant decrease in the C content (C(1 s) in the C-C peak at 285 eV) was observed after DUV irradiation, confirming that the SAM was degraded during the DUV irradiation. At the same time, the Si(2p) (102 eV) peak increased, which is expected if the thickness of the SAM decreases. The depth of analysis of XPS is greater than the SAM initial thickness, so the signal from the substrate is expected to increase with decreasing SAM thickness. This decrease was additionally confirmed using ellipsometry measurements (not shown here), with a trend following the contact angle evolution. Interestingly, the O(1 s) contribution (530 eV) also increased during DUV irradiation. This result can be explained by oxidation of the alkyl chains of the SAM. Indeed, the deconvolution of the C(1 s) peak shown in Fig. 4b confirms the increase of oxidized carbon atoms (C-O, C=O and COO at 286.6 eV, 288.0 eV and 289.2 eV, respectively). The DUV irradiation therefore results in shortening and oxidation of the alkyl chains of the SAM. Oxygen from the air probably participates in this mechanism, either from excited states or via the formation of ozone during DUV irradiation. However, we observed that the SAM degradation still occurred under inert atmosphere (N_2_).

It is observed that among the oxidized C species, C-O is predominant and remains at a constant proportion during irradiation (approximately 60% of all oxidized C species). Interestingly, the ratio of the proportions of C=O and COOR is inverted towards the end of DUV irradiation: at lower doses, the proportion of C=O is higher, whereas that of COOR increases at higher doses, which may be explained by the sequential oxidation of C during DUV irradiation.

A schematic representation of the steps that occur during modification of an HTS SAM under DUV irradiation is shown in Fig. 4c. In the first steps of irradiation, rapid oxidation of the surface CH_3_ is assumed to occur. Then, due to the creation of defects, chain scission begins and proceeds progressively.

One question arising from this scheme is whether there are C chains remaining after long irradiation times (15 J/cm^2^ or more). In other words, is DUV irradiation a suitable process for completely removing the grafted SAM from the surface of the sample, as proposed by Stenger *et al*.^51^? In fact, C species were still observable for doses as high as 20 J/cm^2^, but the level was sufficiently low to be attributed to surface contamination, which inevitably occurs for any substrate in a normal atmosphere. To further confirm the complete removal of SAMs, attempts to regraft another monolayer after DUV irradiation were conducted. A sample of UTS SAM prepared on Si was exposed to DUV irradiation (15 J/cm²) and then immediately immersed in an AHAPS solution to achieve the grafting of a different SAM in the irradiated region.

**Figure 5:**
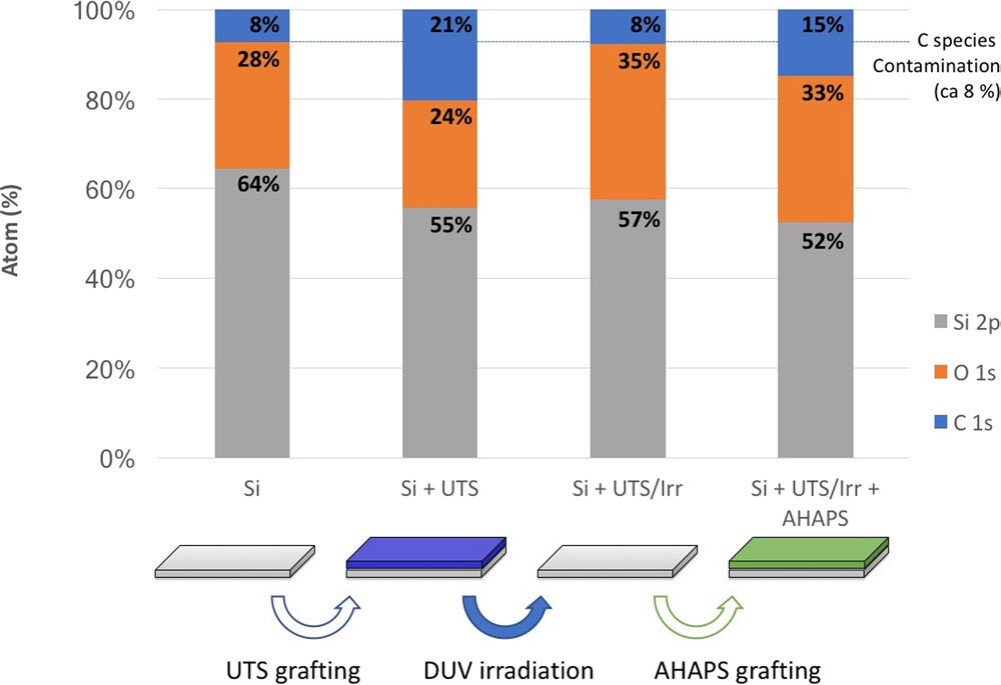
XPS analysis of the surface chemistry of a Si substrate showing the atomic composition of the surface of the Si substrate (i) before and (ii) after UTS grafting, (iii) after DUV irradiation (15 J/cm^2^) and iv) after regrafting of AHAPS on the irradiated substrate. The respective proportions of C, O and Si detected in the analyzed layer are given.

The results of SAM regrafting after DUV irradiation are displayed in Fig. 5. Raw high resolution C(1 s) spectra are given in SI2. They are obtained from XPS analysis of the surface at several stages of the process. The first sample corresponds to a bare Si substrate after cleaning by UV-ozone treatment. Despite extensive UV-ozone cleaning, carbon contamination is observed (8%). This surface contamination is presumed to be present in all samples and can be considered a background signal. After grafting UTS, the content of C increased up to 21%, in good agreement with the expected value for such an SAM. After DUV irradiation, the initial content of C is restored, which can again be attributed to surface contamination. These irradiation conditions thus correspond to a total removal of the SAM. It is worth mentioning that the content of O is increased, similar to the observed content in the case of HTS. Oxidation of the top-most part of the Si substrate could account for this. In the end, this will lead to an increase of the SiOH functions, thus favoring the grafting of the second SAM. The DUV laser irradiation thus promotes the creation of SiOH species, which can favor the regrafting of a second SAM. The rightmost part of Fig. 5 illustrates this phenomenon: an increase in the C content is clearly observed again and corresponds to a grafted AHAPS monolayer. However, the final C content is slightly lower than the detected value for UTS, probably for two reasons. On the one hand, the AHAPS precursor has a shorter alkane chain (9 instead of 12), and on the other hand, the presence of partial remains of the UTS monolayer cannot be excluded, which would alter the grafting of the second SAM. It must be emphasized that the grafting of the AHAPS was followed by a careful washing of the surface as a final step to ensure that only covalently attached precursors remained on the surface. Considering this, we can argue that the regrafting of another SAM after DUV irradiation is possible and efficient.

### SAM Patterning

The principal advantage of radiation-induced modification of surface chemistry is to open the door towards localized chemistry modification via photolithography approaches. The previously described effects can indeed be obtained locally, on the condition that spatial control of the irradiation is achieved. Additionally, DUV lithography allows nanoscale resolution as a consequence of the short wavelengths approaching the diffraction limits. The aim of this section is to illustrate the possibility of obtaining sub-microscale chemistry patterning via DUV irradiation.

**Figure 6:**
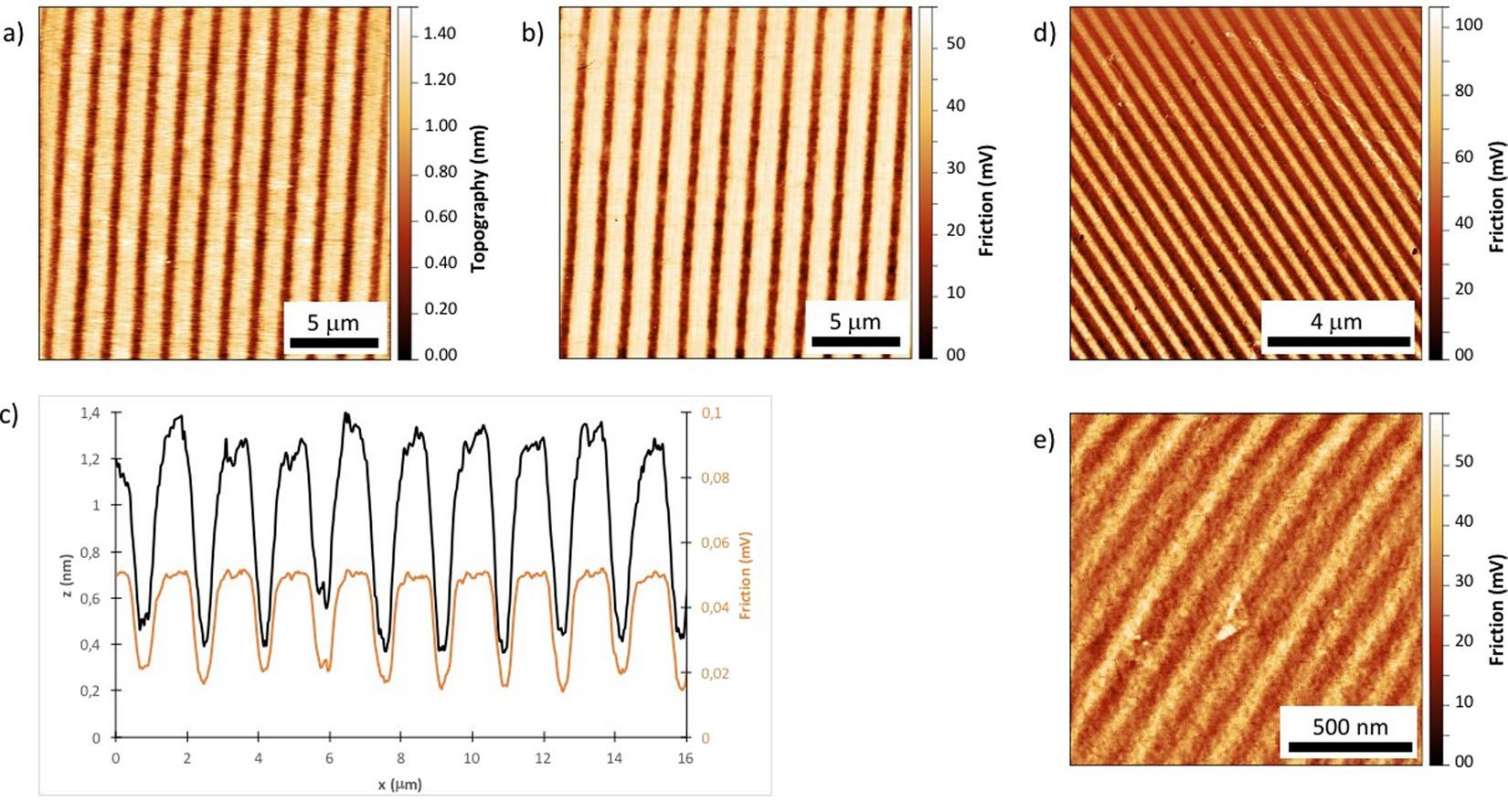
Typical images of chemical patterns obtained by DUV irradiation of an HTS SAM. (**a**–**c**) Topography, friction images and cross sections of the patterned surfaces (binary mask with 0.8 µm line width, 20 J/cm^2^). (**d**,**e**) Friction images of samples patterned by interference lithography with typical pattern widths of 300 nm and 75 nm, respectively, obtained for 30 J/cm^2^.

Figure 6 provides AFM topography and friction images after DUV mask lithography to illustrate DUV patterning with two different irradiation configurations: in Fig. 6a–c, a binary chromium mask (Edmund Optics) was used. This mask has transparent and opaque parts (both parts have the same width, equal to half the period). Because the SAM film is dry and covalently bound, it is not damaged by contact with the mask, which was put in direct contact with the SAM. Under such conditions, diffraction effects are limited, and it can be ensured that the light patterns closely correspond to the mask drawing with a maximum contrast (the mask substrate is fused silica, which is transparent to DUV, and the chromed parts completely block the light).

In Fig. 6d,e, patterns are written by an interference lithography approach: the laser beam is split by a phase mask and recombines at the substrate surface. The interference between the two coherent beams results in a spatially modulated light pattern. A more detailed description of the different experimental setups can be found in a previous publication^38^. The major difference between the two configurations is that the resulting light pattern has a sinusoidal shape in the interferometric case, and as a consequence, there are no completely “dark” regions. In this configuration, the irradiation conditions must be adjusted carefully, as overexposure may provoke the photolysis of the SAM in the dark regions. In contrast, overexposure has fewer consequences when using mask lithography, as the light is blocked in dark regions. Using deep-UV light, an interference approach can generate line widths as low as 50 nm, as shown in a previous study^41^.

The topography images show a typical depth of patterns of 1 nm, which corresponds to the value expected for an HTS monolayer. This further confirms that the DUV irradiation provokes a degradation of the SAM down to the substrate and that there is no degradation of the SAM in dark regions. The period of the patterns perfectly matches the period of the mask used.

In addition to topography, friction images were recorded. The friction value corresponds to the degree of lateral torsion of the AFM cantilever moving across the sample surface. When the tip slides through hydrophilic and hydrophobic regions, the lateral forces applied on the cantilever are thus different, depending on the interactions between the tip and surface. This mode can thus produce detailed images of the chemistry contrast on the sample^52^. The friction images in Fig. 6 show a good contrast between the irradiated and non-irradiated regions, revealing a binary (hydrophobic/hydrophilic) surface chemistry. This contrast is demonstrated with sub-micron resolution in Fig. 6d,e, with hydrophilic and hydrophobic line widths of 300 nm and 75 nm. In the last example, it is noted that the line edge roughness is significantly increased. However, the chemical contrast can be clearly distinguished at this very small size. Close inspection of Fig. 6e reveals that every second line in the friction image is brighter than the others. This can be explained by a 3 beam interference effect due to the non-complete extinction of the O order by the phase mask. This effect has been observed in other material^53^.

**Figure 7:**
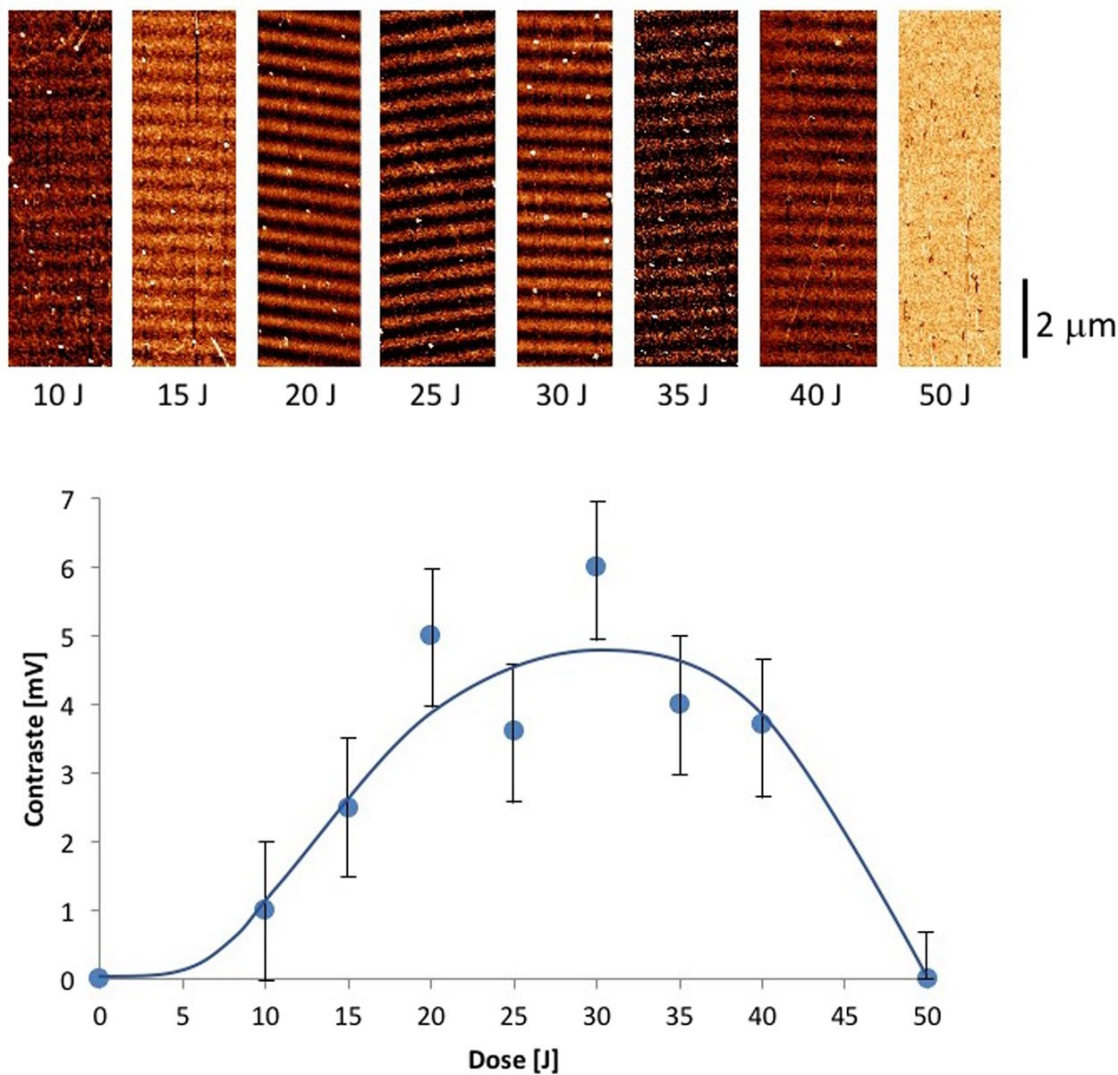
Evolution of the chemical contrast monitored by friction AFM (mV) on the patterns produced by interference irradiation of a UTS SAM (pattern width 300 nm). Top row: AFM images corresponding to each irradiation dose. Bottom: graph illustrating the contrast evolution. The blue line is a guideline for the eye.

Figure 7 illustrates the evolution of the chemical contrast with increasing laser irradiation dose, monitored by AFM in friction mode. An HTS surface was used, and irradiation was conducted in the interference configuration (period of the light pattern: 600 nm). In the first stages of irradiation, the contrast increased as the degradation of the SAM progressed in the bright interference fringes. A maximum contrast was obtained at approximately 30 J (note that this value cannot be compared to the dose delivered in the homogeneous irradiation configuration because some energy is lost in the interferometer, as only two diffracted beams are used). When irradiation was continued beyond this optimum amount, the contrast began to decrease because the photolysis of the SAM proceeded into the darker fringes of the interference pattern. For sufficiently long irradiation times, the chemical contrast disappeared completely, and the surface became homogeneously hydrophilic. These results demonstrate that the non-linear photoresponse of SAMs under DUV irradiation (Fig. 3) is sufficient to obtain a binary chemical response at the nanoscale. DUV interference lithography can thus be used to generate nanometer scale chemical patterns on a wide area (cm^2^ areas are typically achievable in a single exposure) in a single step and within a few minutes of irradiation, which makes this a very efficient and promising approach to obtaining patterned hydrophilic/hydrophobic surfaces.

**Figure 8:**
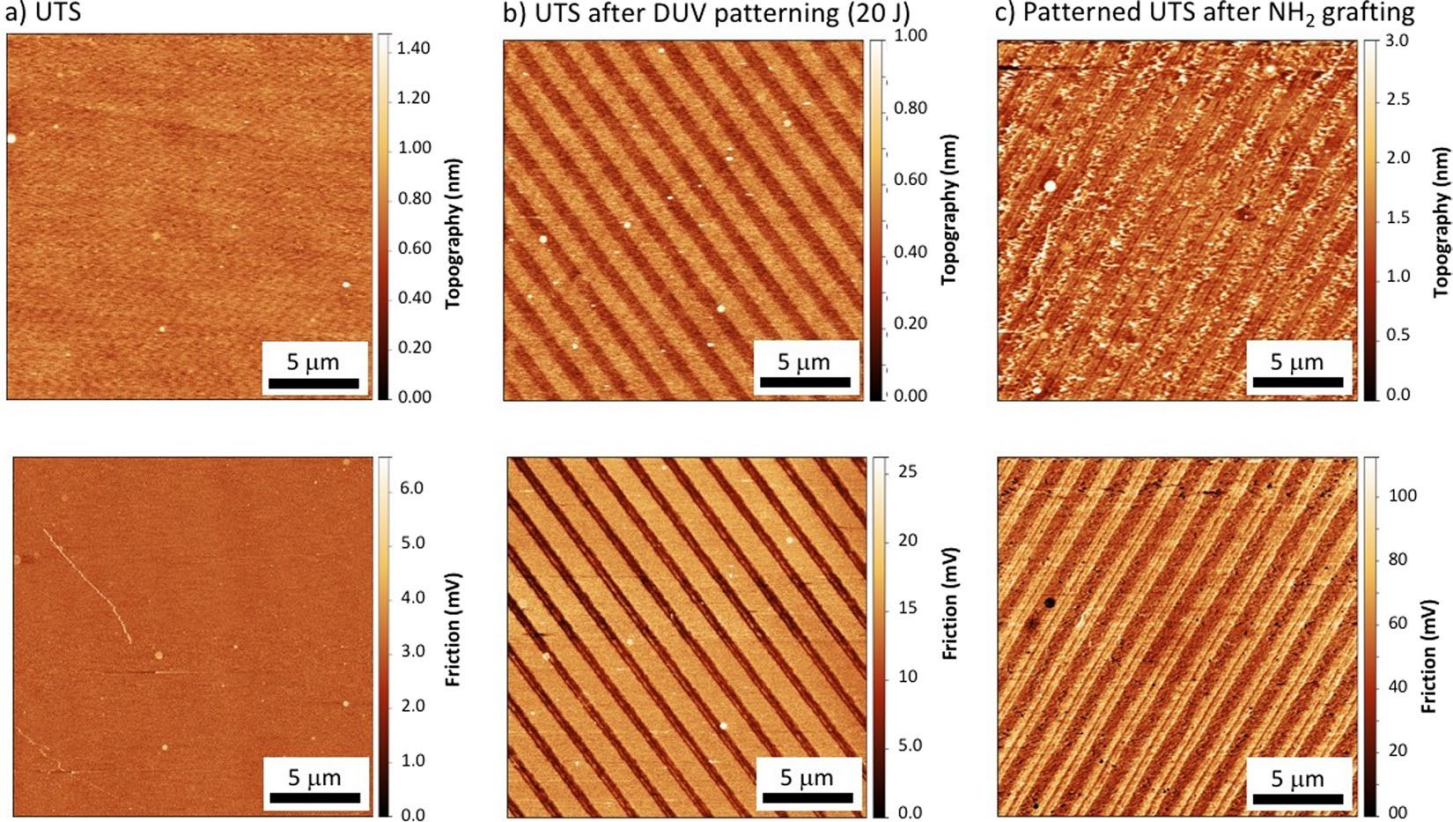
Contact mode AFM images, showing topography (top) and friction (bottom) images of (**a**) a UTS SAM surface, (**b**) UTS SAM surfaces patterned with DUV irradiation (20 J/cm^2^), with a 1.6 μm period chromium mask, and (**c**) the same patterned UTS surfaces after regrafting with an AHAPS precursor.

Even more interesting is the possibility of regrafting a second SAM and then tuning the surface chemistry with submicron resolution, as demonstrated in Fig. 8.

In this example, a silicon wafer was functionalized with UTS and then irradiated with a binary mask (20 J/cm^2^). The AFM image reveals the expected degradation of the SAM in the irradiated regions. Following the procedure previously presented in Fig. 5, the sample was subsequently immersed in another silane solution and then carefully washed to remove any non-grafted molecules. After drying, AFM images were recorded to examine the new surface.

In the first experiment, we used UTS as a second SAM to confirm that regrafting can be efficiently achieved. In this case, the patterns could hardly be distinguished after regrafting, proving the possibility of regrafting with spatial control (not shown), which is of course much more interesting if another SAM is used.

In Fig. 8, we compare the topography and friction images of a) the unmodified UTS SAM surface, (b) the patterned UTS SAM surface, and (c) the same surface after regrafting by an AHAPS precursor. As expected, the surface of the UTS SAM sample was perfectly flat and revealed no surface relief. After DUV irradiation, structuration was obtained as previously described. After regrafting, a modification of the surface was observed: alternating smooth and rough regions appeared in the topography image. The spatial distribution corresponds well to the irradiation pattern, and the chemical contrast can clearly be identified. AHAPS precursors are known to form rougher surfaces, as aggregations of the precursor can hardly be avoided, which then form particles in competition with the surface grafting^48^. The morphology revealed by AFM shows that the process of regrafting AHAPS in the irradiated regions was successful.

Interestingly, an inversion of contrast between topography and friction images was observed: in (b), high topography zones (non-irradiated parts) correspond to the highest friction, whereas in (c), high friction corresponds to low topography. With these results, it is possible to attribute the surface chemistry to both types of regions.

This shows that the degradation of precursors by DUV radiation and the possible spatial resolution yield sufficient results to efficiently obtain dual chemistry patterns with sub-microscale resolution. In the future, even more complex surfaces could be produced, combining more and other precursors and more sophisticated geometries of patterns owing to the use of customized masks or laser direct writing strategies.

### Application to Directed Self-Assembly

As stated in the introduction, the motivation for this work is to provide a convenient and versatile route to obtain substrates with chemical contrast that allows controlled deposition of nano-objects by self-assembly. This last section is aimed at showing some examples in this direction.

This approach combines the advantages of alternative techniques based on photolithography^14–20^, e-beams^21–23^, scanning probe techniques^24–28^ and soft lithography: nanoscale resolution can be obtained in a single step over a relatively wide area (cm^2^). The use of high-energy photons ensures a strong light-matter interaction that allows an interaction with most organic molecules. The patterning of an SAM with any organic function is thus achievable, whereas other photolithography techniques are limited to specific molecules with photoactivatable chromophores. DUV laser beams can be used in air, so no vacuum system is needed, which simplifies the equipment and makes the process cost-effective. The main drawback of this approach may be related to the relatively long irradiation times needed for patterning, which limits the potential industrial applications. However, the single-step process and wide area patterning make it quite competitive versus alternative techniques.

**Figure 9:**
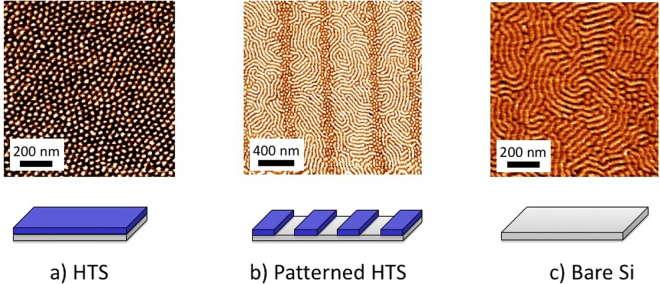
Directed self-assembly of block copolymer revealed by AFM phase images in resonant mode. A PS-b-PMMA solution in toluene was deposited by spin-coating to obtain a film with a thickness of approximately 35 nm. In (**a**), a Si wafer fully covered with HTS was used. It was slightly irradiated (2 J/cm^2^) to avoid dewetting of the polymer film on the HTS surface. In (**b**), photopatterning was conducted through a 1000 nm phase mask (15 J/cm^2^). In (**c**), deposition of the PS-PMMA thin films was performed on a Si substrate.

The first example, depicted in Fig. 9 is related to the DSA of block copolymers. Block copolymers are known to spontaneously self-organize into nanodomains corresponding to each block. Here, PS-b-PMMA (21000 g/mol (PMMA) and 43000 g/mol (PS)) was chosen as a reference system. The molecular weight of the macromolecules and the size of the block lead to self-assembly in a cylindrical system, as predicted by the Flory-Huggins theory^54,55^. Three different surfaces were chosen: (a) an HTS SAM irradiated with a low dose (2 J/cm^2^) to obtain a water contact angle of 90°, (b) an HTS SAM irradiated through a binary mask (15 mJ/cm^2^) to generate hydrophilic/hydrophobic patterns, and (c) a bare Si substrate activated by UV-ozone cleaning. The third surface was completely hydrophilic and can also be obtained by DUV laser irradiation of the Si substrate. On each surface, the block-copolymer solution was deposited by spin-coating to obtain a thickness of approximately 35 nm, evaluated via ellipsometry. The low dose irradiation of the HTS surface was necessary (first case) because the block copolymer thin film could not be deposited on HTS raw film (dewetting of the polymer film was observed). After spin-coating, the films were heated on a hot plate at 170 °C for 60 min to obtain self-organization. On the HTS SAM, a cylindrical organization with an orientation perpendicular to the surface was obtained, whereas on the bare Si substrate, the blocks oriented themselves parallel to the surface.

The orientation of the block copolymer self-assembly is governed by the surface energy of the substrate: if the substrate has affinity to only one of the blocks, this block will preferentially adhere to the substrate and force the second block to organize parallel to the substrate. In contrast, if the substrate affinity to each block is balanced, the system will minimize its energy by organizing the blocks perpendicular to the surface and ensure contact with both blocks.

On the chemically patterned sample, both orientations are obtained. This confirms the ability of DUV patterning to generate the hydrophilic/hydrophobic contrast needed at the microscale and the utility of our fabrication method in this framework. Further works are conducted to optimize the patterning of SAMs and the thermal curing of block copolymer thin films to improve the directional assembly of the patterns. In fact, on patterned surfaces, the horizontally organized domains of block copolymers are expected to align with the pattern direction, which can be useful for applications in microelectronics, data storage or photonics. This route is much more versatile than other processes that have been developed, including those involving electric fields^56,57^, shear alignment^12^, directional crystallization^58^ and thickness control^59^. These processes are intrinsically limited to specific copolymers, which again limits the range of potential applications.

**Figure 10:**
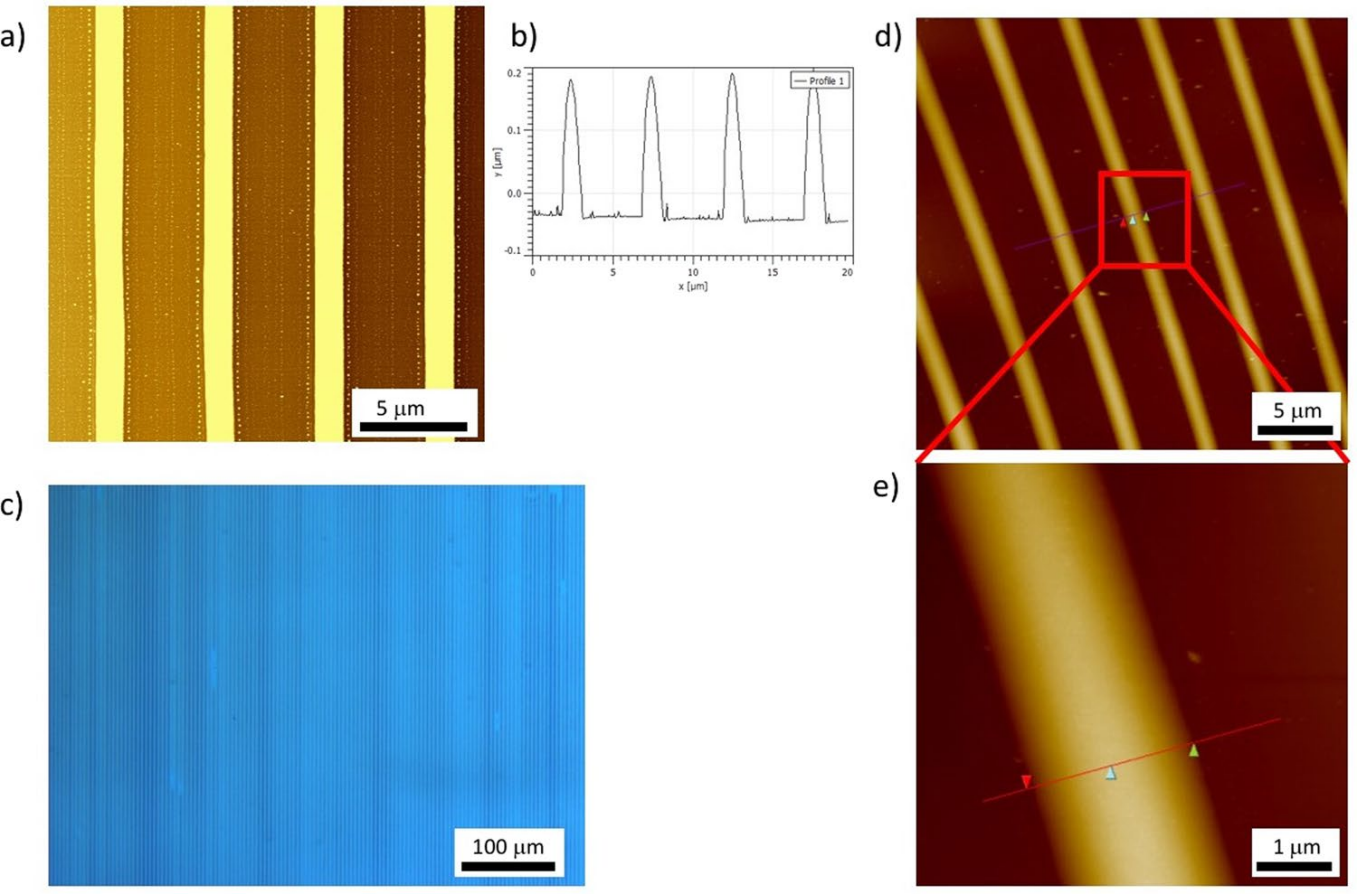
Metal oxide microstructures created by directed dewetting of metal oxide solutions on a DUV patterned surface. (**a**,**b**) AFM topography images and cross-section (contact mode), respectively, of an In precursor solution deposited on a silicon wafer coated with HTS and irradiated through a binary chromium mask (5 μm period) with 15 J/cm^2^. (**c**) Optical microscopy image of the same sample. (**d**,**e**) AFM topography images related to an InGaZnO precursor solution. The surface for deposition was also prepared from an HTS SAM on Si and irradiated through a binary chromium mask (5 μm period) with 15 J/cm^2^.

In a second example, we used solutions of metal oxide precursors to show that the DUV chemically patterned substrates can be used to obtain localized deposition via a dewetting effect. We recently proposed solutions of In^2+^ to prepare InO materials with conductive properties through sol-gel chemistry. The same strategy can be used with In^2+^, Zn^2+^ and Ga^2+^ ions, which leads to InGaZnO materials with semi-conductive properties. Metal oxide materials remain challenging to etch, and microfabrication methods of metal oxide structures with electrical properties have thus been an intense field of research in recent years, primarily for applications in microelectronics. As an alternative microfabrication method, we proposed to spin-coat a thin film of dissolved sol-gel precursors onto a chemically patterned HTS surface. The thin film subsequently undergoes spontaneous dewetting, being thermodynamically unstable. During dewetting, liquid concentrates in the regions with maximum affinity; then, solvent drying occurs, and as condensation reactions proceed, metal oxide microstructures are created. Examples of such structures are presented in Fig. 10.

In Fig. 10a, we can clearly distinguish the InO patterns using AFM after drying the sample. The structures are 1.5 micron wide and approximately 200 nm high, as illustrated in the cross-section (Fig. 10b). In Fig. 10c, we illustrate that a relatively wide area (mm^2^) can be covered with such microstructures when the deposition step can be controlled to produce a homogeneous liquid thin film over the entire region. Similar results can be obtained with other metal oxide precursors. In fact, this method is generally applicable as soon as a precursor solution is prepared, which is possible for almost any metal oxide (or mixture). Although photosensitive metal oxide precursors were recently proposed for direct laser writing^39,40^, some precursors remain very challenging to pattern, such as InGaZnO. Figure 10d,e show patterns of InGaZnO, obtained using the same methods, to demonstrate the versatility of the process. For this last example, it has to be emphasized that because the dewetting process is governed by surface tension phenomena, the resulting surface presents a notable smoothness. This point can be very important for improving the electrical or optical properties of micro- or nanostructures. Finally, we would like to stress that the process was performed on Si substrates, but it could also be applied to other substrates as long as they can be functionalized with a monomolecular layer or their surface chemistry can be modified by DUV irradiation, which, for example, is the case for most polymer surfaces.

After controlled liquid deposition, we show in a last example that these surfaces can also induce localized condensation of water, leading to microdroplets of controlled morphology. First proposed by Whitesides *et al*.^60^, the observation of these so-called breath figures is a convenient way to reveal a chemical contrast on a patterned substrate. In our experiment, HTS SAMs were patterned with binary masks. In a first experiment, a single exposure of 30 J/cm^2^ was performed with a mask with a period of 10 μm. The dose was chosen to ensure complete degradation of the SAM in the irradiated regions, as shown in Fig. 3. This sample is thus composed of 5 μm wide parallel hydrophilic lines separated by 5 μm wide hydrophobic lines. In a second experiment, two irradiation steps, with a 90° rotation of a 5 μm period mask between steps, were used to produce 2.5 µm × 2.5 µm square hydrophobic domains distributed throughout a square hydrophilic lattice continuum. The dose (20 J/cm^2^) also ensured that the HTS SAM was preserved in the square region where no irradiation occurred.

A 10 μm period mask was used on the HTS surface with a DUV irradiation of 30 J/cm^2^. The chemical patterns are thus 5 μm wide hydrophilic lines separated by 5 μm wide hydrophobic lines.

**Figure 11:**
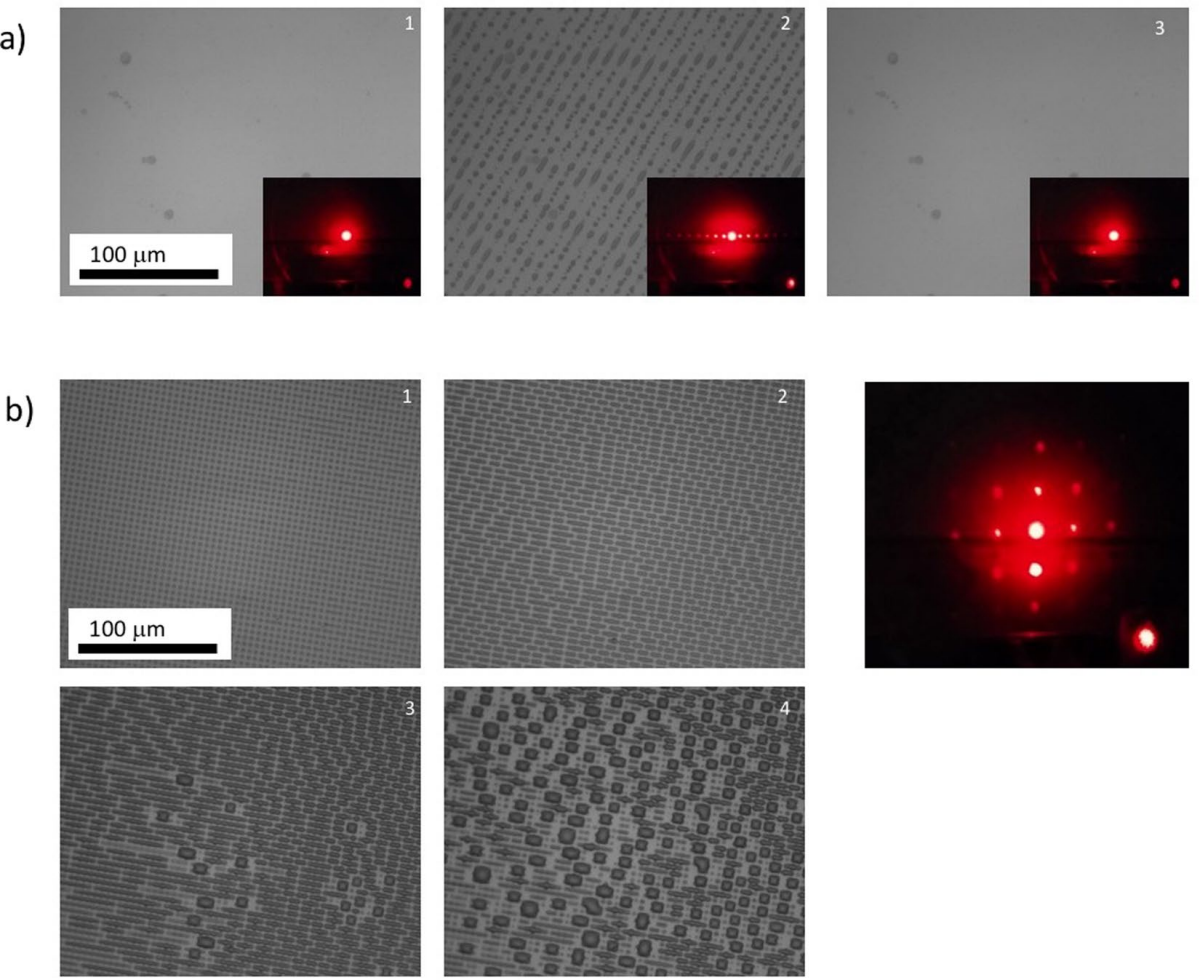
Locally induced condensation of water on a chemically patterned surface. (**a**) A 10 μm period mask was used on an HTS surface with DUV irradiation at 30 J/cm^2^. The chemical patterns are thus 5 μm wide hydrophilic lines separated by 5 μm wide hydrophobic lines. Microscopy images of the same sample, under room humidity conditions (left), under increased humidity (middle), and at the initial humidity content (right). In each case, the insets correspond to the light patterns of a He:Ne laser transmitted through the sample. (**b**) Sample prepared with 2 irradiations (2 × 20 J/cm^2^) with rotation of the 5 μm period mask between the two irradiations, resulting in an array of 2.5 μm × 2.5 μm HTS hydrophobic squares separated by hydrophilic regions (SiOH). The sequence of microscopy images corresponds to increasing humidity in the atmosphere surrounding the sample. A diffraction pattern of a He:Ne laser is also given, confirming the 2D chemical patterning with good reproducibility in the x and y directions.

Figure 11a shows the growth of condensing water droplets on a sample patterned with hydrophilic lines. The microdrops are clearly localized on the hydrophilic lines but do not form continuous segments for energetic reasons. Further condensation led to crossing of the hydrophobic lines by the growing droplets, as already demonstrated^61^. In the case of square patterns (Fig. 11b), microdroplets are first localized at the cross-section between two hydrophilic lines before collapsing and covering the hydrophobic domains, as expected^62^. In both cases, the phenomenon was completely reversible and could be continuously reproduced.

These results demonstrate that the hydrophilic patterns produced by DUV radiation can effectively confine condensed water structures at the micron scale. In a separate experiment, we turned these water condensation patterns into a diffraction grating for a He:Ne laser (633 nm) and used transparent glass microscope slides as substrates. The diffraction patterns obtained with both patterns are shown as insets in Fig. 11a,b. The separation between each diffraction order fully corresponds to the value expected by the dimension of the hydrophilic pattern. Moreover, the diffracted spots exhibit good brightness and a limited amount of diffused light, showing that the liquid gratings have interesting optical properties, despite the non-continuous lines. It also demonstrates that the transient water microstructures can act as a diffraction grating and produce temporary diffraction patterns.

More complex patterns could be produced in the future, such as computer-calculated holograms. Pixel-by-pixel DUV patterning could then be used to record a latent image that could be revealed by increasing the local atmospheric humidity. Light diffraction through such structures could be used to create transient images or messages.

## Conclusion

Chemical patterning using a DUV laser was investigated using mask and interference lithography. This experimental configuration proved to be very efficient and versatile for chemical patterning. Photoinduced modification of the monolayer was characterized by spectroscopy and AFM, and the surface chemistry modification induced by 193 nm laser irradiation was shown to be sufficient to obtain chemical contrast at the submicron scale. The usefulness of such local chemical patterning was illustrated through several examples of directed self-assembly to demonstrate the potential of this process. These proofs of concept will be further explored in the near future.

## Electronic supplementary material

Supplementary Information
