# Supplementary material for: Deep-UV photoinduced chemical patterning at the micro- and nanoscale for directed self-assembly
Source: Sci Rep. 2018 Jul 11;8:10444. doi: 10.1038/s41598-018-28196-1 (PMC6041335; doi:10.1038/s41598-018-28196-1)
Supplement: Supplementary file 1 — Supplementary Information [file 41598_2018_28196_MOESM1_ESM.docx]

**Deep-UV photoinduced chemical patterning at the micro- and nanoscale for directed self-assembly**

Benjamin Leuschel, Agnieszka Gwiazda, Wajdi Heni, Frédéric Diot, Shang-Yu Yu, Clémentine Bidaut, Laurent Vonna, Arnaud Ponche, Hamidou Haidara† and Olivier Soppera*

Institut de Science des Matériaux de Mulhouse, CNRS-UMR 7361.

Université de Haute Alsace, 15 rue Jean Starcky, 68057 Mulhouse, France.

* contact : olivier.soppera@uha.fr,

† in memoriam of Hamidou Haidara who tragically left us on 1^st^ May 2016.

**Supporting information**

**a)**

**b)**

**SI1 :** XPS spectra of HTS SAMs, irradiated a) with 2.5 J/cm^2^ and b) 20 J/cm^2^.

^^

**SI2 :** C1s peaks of the XPS spectra for Si substrate (red), Si functionalized with UTS (blue), Si functionalized with UTS and irradiated (green) and after regrafting with AHAPS (black)
